# Relationship between disease impact scores and C-reactive protein/albumin ratio in patients with psoriatic arthritis

**DOI:** 10.3325/cmj.2022.63.141

**Published:** 2022-04

**Authors:** Tugba Izci Duran, Melih Pamukcu

**Affiliations:** 1 Department of Internal Medicine, Division of Rheumatology, Ondokuz Mayıs University Medical Faculty, Samsun, Turkey; 2 Clinic of Rheumatology, Dışkapı Yıldırım Beyazıt Education and Research Hospital, Health Sciences University, Ankara, Turkey

## Abstract

**Aim:**

To evaluate the relationships between the C-reactive protein (CRP)/albumin ratio (CAR), neutrophil/lymphocyte ratio (NLR), platelet/lymphocyte ratio (PLR), and Disease Activity in Psoriatic Arthritis (DAPSA) and Psoriatic Arthritis Impact of Disease 12-item-questionnaire (PsAID-12) scores in patients with psoriatic arthritis (PsA).

**Methods:**

This cross-sectional study involved 160 (121 female) patients with PsA who were >18 years old and treated in the rheumatology clinic of Dışkapı Yıldırım Beyazıt Education and Research Hospital between January 2020 and January 2021. Demographic and clinical data, PsAID-12 and DAPSA scores, CRP, erythrocyte sedimentation rate (ESR), albumin level, neutrophil, lymphocyte, and platelet counts were recorded.

**Results:**

The mean age was 46.49 ± 11.12 years; median (min-max) disease duration was 2 years (0.5-34). The PsAID score was ≥4 (high disease impact) in 74.4% of patients. Patients with high disease impact had significantly higher CRP, ESR, CAR, NLR, PLR, neutrophil counts, and DAPSA scores (*P* < 0.001). PsAID scores significantly highly correlated with CRP (rho 0.864, *P* < 0.001), DAPSA significantly highly correlated with the CAR (rho 0.890, *P* < 0.001). Receiver operating characteristic curve analysis showed that the CAR (area under the curve [AUC] 0.901, *P* < 0.05, 95% confidence interval [CI] 0.855-0.947, NLR (AUC 0.759, *P* < 0.05, 95% CI 0.680-0.838), and PLR (AUC 0.686, *P* < 0.05, 95% CI 0.591-0.782) predicted high disease impact. The cut-off value for the CAR was 0.98.

**Conclusion:**

The CAR can be useful in daily practice as a simple and quick assessment method to evaluate disease impact in PsA.

Psoriatic arthritis (PsA) is a chronic, immune-mediated inflammatory disease that manifests as peripheral arthritis, enthesitis, dactylitis, spondylitis, and skin and nail psoriasis (1). It has a prevalence rate of 0.01-1%, depending on the geographic region (2,3). The symptoms of PsA negatively affect the patients' health-related quality of life (4-7). Valid and reliable disease activity scales are needed to evaluate the efficacy of increasingly new treatment options and to ensure standardization in clinical trials (8). Therefore, many scoring systems have been developed to evaluate disease activity status and response to medication. These scoring systems evaluate disease activity based on the number of painful and swollen joints involved, the level of inflammatory markers, global assessment of disease activity, and radiographic findings. The Disease Activity in Psoriatic Arthritis (DAPSA), a clinical disease activity index, is a commonly used scale to evaluate disease activity in patients with PsA (9). Another scale is the Psoriatic Arthritis Impact of Disease 12-item questionnaire (PsAID-12), recently developed by the European League Against Rheumatism (10). PsAID has high validity (10,11). The two scales significantly correlate in the evaluation of patients with PsA (9).

In addition to composite scores such as PsAID and DAPSA, there is a need for simple and rapid scales with proven validity and reliability that can be used in outpatient clinic setting. The neutrophil/lymphocyte ratio (NLR) and platelet/lymphocyte ratio (PLR) are markers of systemic inflammation (12,13). Another inflammation marker, the C-reactive protein (CRP)/albumin ratio (CAR), has prognostic significance in inflammatory processes such as cardiovascular diseases and stroke (14,15). The CAR reflects inflammation more accurately than serum CRP level (15,16). Although it is a valuable indicator of systemic inflammation (17,18), the relationship between disease activity and the CAR in PsA has not been investigated. Therefore, this study investigated the relationship between the CAR, NLR, PLR, and DAPSA with PsAID composite score in patients with PsA.

## Patients and methods

This cross-sectional study enrolled 160 patients aged >18 years who were regularly followed up in the rheumatology clinic of Dışkapı Yıldırım Beyazıt Education and Research Hospital between January 2020 and January 2021 and diagnosed with PsA according to the Classification Criteria for Psoriatic Arthritis. Patients with malignancy, other chronic inflammatory diseases, active hepatitis, chronic liver or kidney failure, and active infections, as well as those who were pregnant or breastfeeding were not included in the study.

The study conforms to the Helsinki Declaration principles. The Ethics Committee of Dışkapı Yıldırım Beyazıt Education and Research Hospital issued the ethical approval (103/09).

Demographic and clinical data were recorded. The PsAID and DAPSA scores were calculated. CRP (reference range, 0-5 mg/dL), ESR (reference range, 0-10 mm/h), and albumin (g/dL) levels were obtained based on laboratory analyses. Leukocyte, neutrophil, lymphocyte, and platelet counts (all K/μL) were determined based on complete blood count analysis. The CAR was calculated by dividing CRP level by albumin level. The NLR was calculated by dividing neutrophil count by lymphocyte count. The PLR was calculated by dividing platelet count by lymphocyte count.

The Psoriatic Arthritis Impact of Disease (PsAID) questionnaire is used to assess the impact of PsA from the patients' perspective (10). It is available in two versions: a 9-item and a 12-item questionnaire (10). Each question in PsAID-12 represents a PsA-specific life domain: pain, fatigue, skin problems, work and/or leisure activities, functional capacity, comfort status, sleep disturbances, coping, anxiety, shyness and/or timidity, social engagement, and depression. The effect of symptoms on each domain is rated from 0 to 10. The total score is divided by 20. The final score ranges from 0 (best case) to 10 (worst case), and the cut-off value is 4. A PsAID score of <4 (low disease impact) describes a symptom level acceptable to the patient (10).

DAPSA collects the data on the number of tender and swollen joints, serum CRP level, the patient’s global assessment of the level of disease activity, and pain assessment on a 10-cm visual analog scale (9). A DAPSA score of ≤4 indicates remission and that of ≤14 indicates low disease activity (9).

### Statistical analysis

Data are presented as counts and percentages, mean ± standard deviation, or median (min-max). The normality of distribution of numerical variables was tested with the Shapiro-Wilk test. The independent-samples *t* test and the Mann-Whitney U were used for intergroup comparisons of numerical variables. Categorical data were evaluated with the χ^2^ test. Spearman correlation analysis (rho) was used to assess the correlation between the variables. Correlation 0.05-0.30 was considered as low, 0.30-0.40 as low-moderate, 0.40-0.60 as moderate, 0.60-0.70 as good, 0.70-0.75 as very good, and 0.75-1.00 as high (19). Variables that were correlated with high disease impact according to PsAID were analyzed with receiver operating characteristic (ROC) analysis. Area under the curve (AUC) was calculated. The cut-off value was determined with the Youden index (Youden index = sensitivity + specificity - 1) as it is the cut-off value with the highest AUC and aims to maximize the difference between the true positive rate and the false positive rate. A *P* value of <0.05 was considered significant. Statistical analysis was performed with SPSS, version 22.0 (IBM Corp., Armonk, NY, USA).

## Results

The study enrolled 160 patients (121 female, Table 1). The mean age ± standard deviation was 46.49 ± 11.12 years, and the median (min-max) disease duration was 2 (0.5-34) years. Among these, 122 (76.3%) patients diagnosed with PsA had previously been diagnosed with psoriasis.

**Table 1:** Characteristics of patients with psoriatic arthritis. Unless otherwise stated, values are presented as median (min-max)*

Parameter	Patients (n = 160)
Age, years (mean ± standard deviation)	46.49 ± 11.12
Female, n (%)	121 (75.6)
Disease duration, years	2 (0.5-34)
Presence of psoriasis, n (%)	122 (76.3)
Psoriatic Arthritis Impact of Disease 0-10	5.55 (1.05-9.65)
Treatment, n (%)	
NSAIDs	15 (9.4)
NSAIDs + csDMARDs	114 (71.3)
bDMARDs	17 (10.6)
csDMARDs + bDMARDs	14 (8.8)
Disease Activity Index for Psoriatic Arthritis	21.91 (10-129)
CRP, mg/L	4.67 (0.12-82.77)
Erythrocyte sedimentation rate, mm/h	10 (2-52)
CAR	1.01 (0.03-19.61)
Neutrophil/lymphocyte ratio	2.41 (0.73-7.10)
Platelet/lymphocyte ratio	134.55 (47.47-492.16)
Hemoglobin, g/dL (mean ± SD)	13.61 ± 1.72
Sacroiliitis, n (%)	
bilateral	52 (32.5)
right	36 (22.5)
left	24 (15)
absence	48 (30)

*Abbreviations: bDMARDs – biological disease-modifying antirheumatic drugs; CAR – CRP/albumin ratio; CRP – C-reactive protein; csDMARDs – conventional disease-modifying antirheumatic drugs; NSAIDs – non-steroidal anti-inflammatory drugs.

Overall, 119 (74.4%) had a PsAID-12 score of ≥4 (high disease impact). Patients with high disease impact had significantly higher CRP, ESR, CAR, NLR, PLR, neutrophil count, albumin level, and DAPSA scores (Table 2). Patients with high disease impact and those with low disease impact did not significantly differ in CRP, albumin, and CAR (Table 3).

**Table 2:** Differences between groups with high and low disease impact according to Psoriatic Arthritis Impact of Disease (PsAID) score. Unless otherwise stated, values are presented as median (min-max)*

	Low disease impact, PsAID<4 (n = 41)	High disease impact, PsAID≥4 (n = 119)	p†
Age, years (mean ± SD)	47.32 ± 12.14	46.20 ± 10.79	0.581
Sex, F/M (n)	27/14	94/25	0.091
Presence of psoriasis, n (%)	34 (82.9)	88 (73.9)	0.292
Treatment, n (%)			0.279
NSAIDs	4 (9.8)	11 (9.2)	>0.999
NSAIDs + csDMARDs	25 (61.0)	89(74.8)	0.110
bDMARDs	6 (14.6)	11 (9.2)	0.380
csDMARDs + bDMARDs	6 (14.6)	8 (6.7)	0.195
Disease duration, years	2 (0.5-34)	2 (0.5-23)	0.741
CRP, mg/L	1.65 (0.12-11.77)	6.2 (0.46-82.77)	<0.001
Erythrocyte sedimentation rate, mm/h	5 (2-24)	13 (2-52)	<0.001
Neutrophil, K/uL (mean ± SD)	4.51 ± 1.65	5.76 ± 1.7	<0.001
Lymphocyte, K/uL	2.30 (1.32-5.15)	2.12 (0.88-4.48)	0.066
Platelet, K/uL	269 (125-446)	286 (156-533)	0.134
Albumin, g/dL (mean ± SD)	4.59 ± 0.22	4.41 ± 0.26	<0.001
CAR	0.35 (0.03-1.02)	1.39 (0.1-19.61)	<0.001
Neutrophil/lymphocyte ratio	1.82 (0.73-3.74)	2.70 (0.85-7.10)	<0.001
Platelet/lymphocyte ratio	108.13 (62.52-211.46)	138.39 (47.47-492.16)	<0.001
Disease Activity Index for Psoriatic Arthritis	15.5 (10-28)	30.32 (11-129)	<0.001

*Abbreviations: SD – standard deviation; bDMARDs – biological disease-modifying antirheumatic drugs; CAR – CRP/albumin ratio; CRP – C-reactive protein; csDMARDs – conventional disease-modifying antirheumatic drugs; NSAIDs – non-steroidal anti-inflammatory drugs.

†Numerical variables were evaluated with the independent-samples *t* test and the Mann-Whitney U test. Categorical data were evaluated with the χ^2^ test.

**Table 3:** Differences between patients according to the presence of psoriasis. Unless otherwise stated, values are presented as median (min-max)*

	Psoriasis		
	yes (n = 122)	no (n = 38)	p^†^
Age, years (mean ± SD)	46.39 ± 10.90	46.79 ± 11.94	0.776
Sex, F/M (n)	88/34	33/5	0.083
Disease duration, years	2 (0.5-34)	2 (0.5-12)	0.458
Treatment, n (%)			0.411
NSAIDs	14 (11.5)	14 (11.8)	0.122
NSAIDs + csDMARDs	84 (68.9)	30 (78.9)	0.305
bDMARDs	13 (10.7)	4 (10.5)	>0.999
csDMARDs + bDMARDs	11 (9.0)	3 (7.9)	>0.999
CRP, mg/L	4.36 (0.31-82.77)	6.0 (0.48-48.07)	0.143
Erythrocyte sedimentation rate, mm/h	10 (2-52)	13 (2-39)	**0.037**
Neutrophil, K/uL (mean ± SD)	5.36 ± 1.86	5.69 ± 1.42	0.208
Lymphocyte, K/uL	2.21 (0.94-5.15)	1.92 (0.88-4.15)	**0.002**
Platelet, K/uL	280 (153-533)	285 (125-478)	0.808
Albumin, g/dL (mean ± SD)	4.46 ± 0.25	4.46 ± 0.30	0.880
CAR	0.96 (0.06-19.61)	1.31 (0.03-11.78)	0.136
Neutrophil/lymphocyte ratio	2.17 (0.73-5.99)	2.91 (1.14-7.10)	**<0.001**
Platelet/lymphocyte ratio	124.10 (50.14-492.16)	153.55 (47.47-311.11)	**<0.011**
Disease Activity Index for Psoriatic Arthritis	19.77 (11-129)	28.19 (10-76)	0.065
Psoriatic Arthritis Impact of Disease (0-10)	5.40 (1.05-9.65)	6.95 (2.40-9.50)	0.104

*Abbreviations: SD – standard deviation; bDMARDs – biological disease-modifying antirheumatic drugs; CAR – CRP/albumin ratio; CRP – C-reactive protein; csDMARDs – conventional disease-modifying antirheumatic drugs; NSAIDs – non-steroidal anti-inflammatory drugs.

†independent-samples *t* test and the Mann-Whitney U.

PsAID-12 scores significantly positive correlated with ESR, CRP, CAR, NLR, PLR, and DAPSA scores. DAPSA scores significantly highly correlated with CAR (rho 0.890, *P* < 0.001) and CAR significantly highly correlated with CRP (rho 0.998, *P* < 0.001). A low-moderate correlation was observed between CAR and ESR (rho 0.572, *P* < 0.001), and between CAR and NLR (rho 0.671, *P* = 0.001, Table 4).

**Table 4:** Spearman correlation analysis between Psoriatic Arthritis Impact of Disease score and clinical and laboratory parameters showing disease activity*

	Rho value	p
C-reactive protein	0.864	<0.001
Erythrocyte sedimentation rate	0.546	<0.001
C-reactive protein/albumin ratio	0.866	<0.001
Neutrophil/lymphocyte ratio	0.592	<0.001
Platelet/lymphocyte ratio	0.357	<0.001
Disease Activity Index for Psoriatic Arthritis	0.758	<0.001

**Figure 1:**
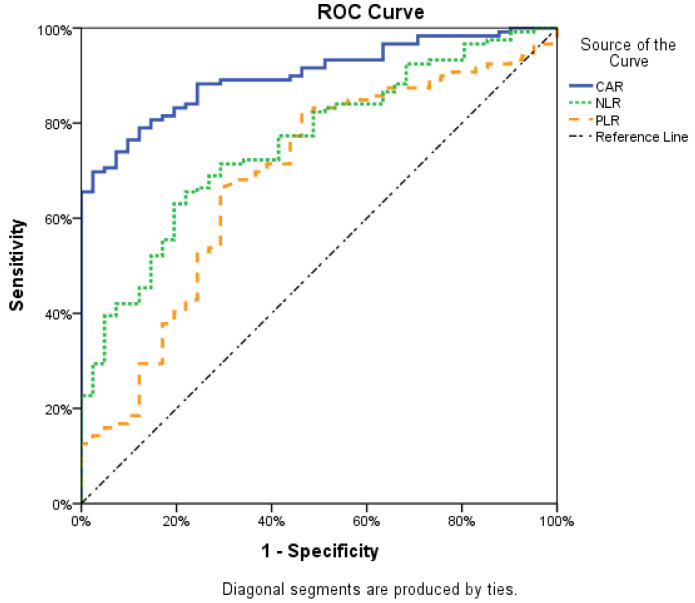
The power of the C reactive protein/albumin ratio (CAR), neutrophil/lymphocyte ratio (NLR), and platelet/lymphocyte ratio (PLR) values to predict high Psoriatic Arthritis Impact of Disease score. Diagonal segments are produced by ties.

The CAR (AUC 0.901, *P* < 0.05, 95% confidence interval [CI] 0.855-0.947), NLR (AUC 0.759, *P* < 0.05, 95% CI 0.680-0.838), and PLR (AUC 0.686, *P* < 0.005, 95% CI 0.591-0.782) were predictors of high disease impact on PsAID-12 (Figure 1). The CAR more effectively predicted high disease impact than NLR and PLR. Using the Youden index, we calculated the cut-off value for CAR to be 0.98. When CAR values above the cut-off value were classified as “high disease impact,” the sensitivity and specificity for CAR were 0.70 and 0.98, respectively (Table 5).

**Table 5:** Area under the receiver operating characteristic curve (AUC), sensitivity and specificity when applying the optimized cut-off points for the C reactive protein/albumin ratio, neutrophil/lymphocyte ratio, and platelet/lymphocyte ratio in predicting high psoriatic arthritis impact of disease

	AUC (95% confidence interval)	Cut-off according to Youden’s Index	Sensitivity (%)	Specificity (%)
C reactive protein/albumin ratio	0.901 (0.855-0.947)	0.98	70	98
Neutrophil/lymphocyte ratio	0.759 (0.680-0.838)	2.30	66	78
Platelet/lymphocyte ratio	0.686 (0.591-0.782)	120.64	66	71

## Discussion

In this study, which investigated the relationship between the CAR and PsAID-12 scores for the first time, the CAR significantly positively correlated with PsAID-12 and DAPSA scores, ESR, CRP, and NLR. The CAR was more closely associated with higher disease impact than the NLR and PLR. In previous studies, patients with PsA had significantly higher NLR and PLR than controls (18,20). Although no previous studies evaluated the relationship between the CAR and PsAID-12 scores in PsA, some studies showed an increased CAR in patients with cancer and those in intensive care, as well as the association of CAR with inflammation (21-23). In addition, in a study by Bozkurt et al. (24) on 35 patients with uveitis episodes and 35 healthy controls, the CAR was an important indicator of uveitis activation. However, few studies investigated acute phase reactants and the CAR in autoimmune diseases. In a recent retrospective study on 32 patients with Takayasu’s arteritis and 32 healthy controls, the CAR was significantly associated with disease activity, CRP, and ESR (16). In a study that included patients with antineutrophil cytoplasmic antibody-associated vasculitis, the CAR at diagnosis was an independent predictor of all-cause mortality (25). In patients with rheumatoid arthritis, the CAR correlated with Disease Activity Score in 28 joints and was used as an indicator of the activity of rheumatoid arthritis (26). Similarly, Sunar et al reported a positive but weak correlation between the CAR and disease activity score in 28 joints-ESR and ESR (17). In another study on patients with rheumatoid arthritis, the CAR significantly positively correlated with CRP and ESR (27). A study on patients with psoriasis reported that the CAR could be used as an inflammatory biomarker of PsA in addition to being an inflammatory biomarker of psoriasis in patients with psoriasis treated with biological agents (28).

Composite indices used to evaluate disease activity in PsA, such as PsAID-12 and DAPSA, have been developed for clinical trials and routine practice. Their disadvantages involve their being time-consuming in daily outpatient clinic settings and containing subjective items evaluated by patients and/or physicians. The CAR is a quick and simple assessment method that positively correlates with PsAID-12 and DAPSA scores. In addition, it more successfully detected high disease impact (PsAID-12 score ≥4) than the NLR and PLR. However, the limitations of our study are a cross-sectional design, lack of a control group, single-center setting, and a relatively small patient group. Another limitation is that PsAID is not only an index of disease activity, but also includes items related to quality of life and functional status. Therefore, in addition to comorbidities, PSAID may be high as a result of irreversible structural damage in psoriatic joints (and adjacent/related structures).

In conclusion, emerging new treatment modalities increase the need for standard measurement methods that objectively assess PsA activity, can be applied easily, and are not time consuming. This study found the CAR to be a practical measurement method as it can be quickly used in routine outpatient settings, does not contain a subjective parameter, is inexpensive, and correlates with validated assessment methods such as PsAID-12 and DAPSA.
